# Patient experiences of consulting advanced practice nurses in primary care: a cross-sectional study

**DOI:** 10.1186/s12913-026-14511-4

**Published:** 2026-04-11

**Authors:** Ruwang Han, Auvo Rauhala, Melanie Rogers, Lisbeth Fagerström

**Affiliations:** 1 Department of Natural and Health Sciences, Faculty of Science and Engineering, Åbo Akademi University, Rantakatu 2, Vaasa, 65100 Finland; 2 Finnish Centre for Client and Patient Safety, Wellbeing Services County of Ostrobothnia, Vaasa, Finland; 3 Department of Nursing, University of Huddersfield, Huddersfield, UK

**Keywords:** Advanced practice nursing, Primary care, Delivery of health care, Patient satisfaction, Patient self-management, Nurse Practitioner, Non-medical prescribing, Cross-sectional study

## Abstract

**Background:**

Advanced practice nursing has been implemented across the world to meet primary care needs and improve care provision. This study had two-fold aims: first, to explore patients’ experiences in terms of patient satisfaction and patient enablement after consulting Finnish Advanced Practice Nurses (APNs) in primary care; second, to explore the potential factors associated with these patient experiences, such as patients’ overall health status and language differences between the APNs and the patients.

**Methods:**

A cross-sectional design was applied in this study, utilising a validated instrument, the Patient Enablement and Satisfaction Survey for Advanced Nurse Practitioners, along with a short questionnaire tailored for APNs. In total, 25 APNs and 483 patients participated in the data collection between January and June 2025. Descriptive statistics, Chi-square test, Fisher’s exact test, independent samples Welch’s t-test, Mann-Whitney U test, one-way Welch’s Analysis of Variance (ANOVA), one-way Kruskal-Wallis test, and Spearman’s correlation were used in the analysis.

**Results:**

Patients highly rated their consultation with APNs in primary care, with high levels of patient satisfaction and enablement being reported. However, slight differences were observed regarding these aspects among Nurse Prescribers, Nurse Practitioners (NPs), and NPs with prescriptive authority. Patients who spoke the same language as the APN and rated their overall health status more positively identified increased satisfaction and enablement regarding APN consultations.

**Conclusions:**

This study provides new evidence of patient experiences in utilising care and services provided by APNs in Finland. It also offers more information for policy makers and nurse leaders on the implementation and overall importance of advanced practice nursing in primary care. The results may also contribute to facilitating the legislative process of registering Finnish NPs and Nurse Prescribers as titles at the national level.

**Trial registration:**

Clinical trial number: Not applicable.

**Supplementary Information:**

The online version contains supplementary material available at 10.1186/s12913-026-14511-4.

## Background

As unmet primary care needs continue to increase across the world, Advanced Practice Nurses (APNs) have been increasingly utilised to improve care provision and meet healthcare demands. On a global scale, the APN workforce continues to evolve in primary care [1]. APNs have been found to substantially contribute to chronic disease management and the provision of emergency care [2, 3]. The implementation of advanced practice nursing has resulted in increased access to primary care, reductions in waiting times and costs, improved patient satisfaction, and higher quality of care [4–6]. Moreover, primary care APNs, including Nurse Practitioners (NPs), play a crucial role in providing safe, effective care and improving healthcare efficiency [1, 7]. This study focuses on patients’ perspectives of APN consultations, including patient satisfaction and patient enablement in Finland.

The development of advanced practice nursing, including NP and Clinical Nurse Specialist (CNS) roles [8], varies internationally. In recent years, a growing gap has emerged among Organisation for Economic Co-operation and Development (OECD) [9] countries regarding the utilisation of advanced practice nursing in primary care. Countries such as the United States and Canada have made considerable progress in advanced practice nursing, while others continue to lag behind. Many countries are still debating the benefits, outcomes, and drawbacks of advanced practice nursing or are in the early stages of implementation [9].

Generally, advanced practice nursing has exerted a positive impact on patient satisfaction [10, 11], which is regarded as a central outcome metric for demonstrating and evaluating NP care. Satisfaction is a variable related to the organisation’s “market share” of clients [12]. In primary care, international research regarding the quality of care provided by NPs has proven to be consistent from both the patients’ and professionals’ perspectives, confirming that better access to healthcare has been achieved in such contexts [13]. In relation to patient experiences, APNs contribute to building strong relationships with patients by emphasising trust and satisfaction [4, 14]. Patient satisfaction is defined in terms of “ how patients evaluate the quality of their healthcare experience” [15]. Accordingly, patient satisfaction within primary care service is associated with individual factors such as age, gender, ethnicity, deprivation, and health status [16]. In one Australian study, over 90% of patients identified that they were satisfied with the care delivered by NPs, especially when NPs took time to talk to them. Patients also reported that NPs were thorough and trustworthy [17]. Similarly, in Ireland, 94.1% (*n* = 80) of patients who answered a patient satisfaction questionnaire reported that the care they received from APNs had met their expectations [18]. Another study in Switzerland showed that patients attending as outpatients had less anxiety and greater satisfaction when consulting with APNs [19]. Overall, these findings confirm that APNs contribute to optimising care delivery and increasing patient satisfaction.

Patient enablement, described as “the patient’s ability to understand and cope with illness after a consultation” [20], is another concept to measuring patient experiences in health care settings [21]. Patient enablement is also regarded as “an indication of patients’ perceptions of their ability, confidence, and empowerment in managing their health care needs” [22]. One particular study demonstrated that patients expressed a higher level of enablement after consulting NPs [23]. Through a holistic consultation approach, NPs are able to enable patients by effectively using their consultation time and building a relationship [21]. By listening to the patients, NP care allows them to be heard and feel that their special concerns have been addressed, further fostering patient enablement [24].

Primary care APN roles in Finland include NPs and Nurse Prescribers [25]. APN education can be undertaken at the University of Applied Sciences (90 credits) or Åbo Akademi University (120 credits). Nurse prescribers are registered nurses (RNs) who have undertaken a separate training programme (45 credits; European Qualification Framework [EQF] – Level 7), which is not included in APN education at the master’s level. Nevertheless, nurse prescribers still obtain the relevant/core clinical skills to practice at an advanced level. Some NPs have also received additional nurse prescribing education. However, neither APNs nor nurse prescribers in Finland have title protection at the national level, which may affect the implementation and development of advanced practice.

The international trend of implementing advanced practice nursing in primary care continues to evolve with the aim of improving access to and the availability of care, treatment, and services. Existing studies have shown that most patients are satisfied with their consultations with APNs. However, in countries where advanced practice nursing is still under development, it is necessary to obtain more evidence of the benefits and patients’ perceptions of APN consultations. After 25 years of APN role development in Finland, very few studies have examined the outcomes of these roles [26]. There is currently no available evidence regarding patients’ experiences, including patient satisfaction and enablement of APN consultations in primary care in Finland.

## Methods

### Aim

This study had two-fold aims: first, to explore patients’ experiences in terms of patient satisfaction and patient enablement after consulting Finnish APNs in primary care and, second, to explore the potential factors associated with these patient experiences, such as patients’ overall health status and language differences between APNs and patients.

### Study design and setting

The cross-sectional design of this study adheres to the STrengthening the Reporting of OBservational studies in Epidemiology (STROBE) guidelines [27]. The study was conducted in a wellbeing services county in Finland, with both Swedish-speaking and Finnish-speaking populations. In 2023, all of Finland was divided into 21 wellbeing services counties. Each county is self-governing and provides healthcare, social welfare, and rescue services for its population. Finnish primary care services are usually provided in social and healthcare centres. Eight social and healthcare centres that offer APN clinics in eight municipalities were included in this study.

### Participants

The study consisted of two separate populations: primary care APNs and patients who had consulted them. In this study, APNs included nurse prescribers, NPs, and NPs with prescriptive authority. The inclusion criteria for APN participants were RNs who had taken APN education (90 or 120 credits) and/or nurse prescribing education (45 credits). A total of 30 APNs working in regional primary care settings were invited to participate in this study. However, five of the APNs declined to participate due to their work being restricted to the home hospital setting (*N* = 1), undertaking only administrative work tasks (*N* = 2), only providing digital services to primary care patients (*N* = 1), and an increased workload (*N* = 1). The primary care APNs in this study were responsible for different patient populations and providing corresponding services and care, including clinics for both long-term conditions and follow-up and health check-up (*N* = 7), acute care services (*N* = 9), and clinics for any health problems (*N* = 9); for more information regarding APN practice patterns, see [3].

The inclusion criteria for patient participants included those who had physically consulted primary care APNs, including patients across all ages (i.e., children, adults, and the elderly). However, patients who only used digital services from APN clinics, such as telephone counselling, were excluded. A total of 483 patients agreed to participate in this study when APNs invited them, but only 425 patients returned the questionnaires after consulting APNs. The response rate of the patient surveys was 425/483 = 88.0%; however, the response rate of the APNs was 483 (100.0%). According to the power analysis, the sample size of patient participants required for one-way Analysis of Variance (ANOVA) with three APN groups was 159, with a medium effect size, alpha of 0.05, and power of 0.80.

### Patient survey and short questionnaire for APNs

An existing validated survey, Patient Enablement and Satisfaction Survey, to evaluate the care provided by Advanced Nurse Practitioners (PESS-ANP) [28] was used in this study. The PESS-ANP is a patient-reported experience measure (PREM) that has been used in Ireland. The original version of PESS [29] has its origins in two validated instruments: Client Satisfaction Tool (CST) [30] and the Patient Enablement Instrument (PEI) [31]. The PESS was originally developed to evaluate patients’ experiences of nursing care in general practice in Australia in 2014 [29].

Specifically, the PESS-ANP includes two sections: 15 statements about patient satisfaction, and five statements about patient enablement [28]. Statements in the first section are measured along a Likert scale ranging from 1 to 5: “1” means “strongly disagree”, “2” means “disagree”, “3” means “uncertain”, “4” means “agree”, and “5” means “strongly agree”. The Likert scale for the second section ranges from 1 to 3: “1” means “same or less”, “2” means “better”, and “3” means “much better”. In addition, there are questions regarding patient background, including gender, age, and overall health status (with options including “very poor”, “poor”, “fair”, “good”, “very good”, and “excellent”). There was also one open-ended question asking why they had visited the social and healthcare centres.

As Finnish and Swedish are the official languages in Finland, the PESS-ANP was translated into these languages. The translation of the PESS-ANP was conducted using professional translation services. To ensure accuracy and quality of translation, cultural sensitivity and adaptation were considered in the translation process. The translation process [32, 33] included forward translation, negotiated consensus between the researchers and translators, back translation, and expert panel discussion among researchers, nurse leaders, and APNs. First, forward translation was conducted through two independent professional translators when translating PESS-ANP into Finnish and Swedish. Second, the translated versions were discussed between the researchers and the translators several times until a consensus was reached. After this, the Swedish and Finnish versions were back-translated into English by two other professional translators. Then, the feedback and results about back translation were sent to the author team.

In the next stage, a face validity approach with one expert panel was used to assess the clarity and conceptual equivalence of the translated versions of the PESS-ANP. The research project group, consisting of three researchers, two NPs with prescriptive authority, and three nurse leaders, went through these translations together to ensure the accuracy of the concepts in both languages, as well as to confirm that the results were understandable and easy to implement in the clinical settings. Finally, the term “patient enablement” was deleted from the translated versions, as there was no suitable Swedish word for “enablement’’. As the APN, NP, or nurse prescribers are not familiar terms to patients in Finland, ‘‘nurse’’ was used instead of these terms in the translated versions. However, patients were informed that nurses in this study included those who worked at an advanced level (i.e., NPs, NPs with prescriptive authority, and nurse prescribers). The translated surveys were pilot tested by two nurses. Additionally, to increase the feasibility of these surveys, they were presented to all APNs working in primary care during three informational meetings (November 2024).

In addition, the APNs answered a short questionnaire [see Additional file 1], including four questions about patient consultations: (1) the length of the consultation time (in minutes) and the administrative time after the consultation (in minutes); (2) the reason for the patient consultation, with options including acute health problems, long-term health problems, or follow-up/ health check-up and an additional open-ended question asking them describe these problems with a few words; (3) whether a physician needed to be consulted during or immediately after the patient visit (yes or no); and (4) whether the patient was referred directly to the physician, directly home without a new appointment, directly home with a new appointment with an APN, directly home with a new appointment with the physician, or for a follow-up with the APN via telephone or videoconference. These questions were available in Swedish and Finnish and were piloted by two NPs with prescriptive authority.

### Data collection

From January to June 2025, the first and fourth authors collected the completed printed surveys. The APNs informed the first author of which language they preferred to use before the hard copies of the short questionnaires were printed. Patients were recruited by APNs and had a choice of Finnish or Swedish in which to answer the survey. The expected time for patients to complete the PESS-ANP was about seven minutes, while the estimated time for APNs to answer the short questionnaires was approximately three minutes. Patients were given information about data storage and protection and the use of data for publications before their participation. In the initial phase, approximately 600 printed surveys and 20 boxes for returned surveys were delivered to APN clinics in person. Each patient survey was assigned an anonymised code linked to the APN’s short questionnaires for the purpose of combining both datasets in the analysis. These codes did not contain any identifiable information and could not be tracked to any individual. Some patients could not answer the surveys by themselves, such as children and very old patients, in which case they could be helped by family members. One APN excluded some patients with multi-morbidities from participating, as the APN felt they were too unwell to accurately and effectively answer the survey. The APNs answered a short questionnaire immediately after each patient visit. Meanwhile, the patients answered their surveys and returned them to a box outside the APN clinic.

### Data analysis

The data were analysed primarily using jamovi software (Version 2.6.44, 2025) [34]. Additionally, statistical software R (version 4.5.2) [35] was utilised for analysing Little’s test with the naniar package [36, 37], and multiple imputation (MI) using the multivariate imputation by chained equations (mice) package [38]. The variables used in this study included patient gender, age, overall health status, consultation duration, administrative work time after consultation, post-consultation care pathways, and the APN’s need to consult a physician. Patients were categorised into three age groups: 0–17 years old, 18–65 years old, and over 65 years old, which correspond to the stages of childhood, adulthood [39], and seniority in the Finnish context. The open-ended question responses were not analysed in this study, as they will be used for another study. Moreover, several variables were added: the total score of all PESS-ANP items, the total score of patient satisfaction items, the total score of patient enablement items, and the language difference between the patient and the APN. The answers regarding the patient’s overall health status were transformed into an inverted scale, with patients in better overall health status having higher scores.

To analyse possible participation bias and its consequences, the responses of the nurses were compared between patients who completed the questionnaire and those who did not participate. Little’s test of missing completely at random (MCAR) was conducted for PESS-ANP items, and the missing data were not MCAR (*p* = <.001). Row mean imputation (RMI) [40] and MI with mice [38] were applied to check the differences regarding the mean and the standard deviation (SD) of the total scores of the PESS-ANP, patient satisfaction, and patient enablement after imputation. When using RMI, the PESS-ANP items were imputed with a maximum of 20% missing data for patient satisfaction items (items 1–15) and patient enablement items (items 16–20). However, among all the PESS-ANP items, the patient satisfaction item scores were very similar between individual respondents (i.e., rows), as were the patient enablement item scores. For example, the SD of the 15 patient satisfaction items for individual respondents was only 0.3, and the response was the same (i.e., mode) for an average of 12.4 items out of 15. This supported the idea that RMI would be well suited for the limited imputation of individual missing patient satisfaction and enablement items in this study. MI was used with the mice package, in addition to complete cases analysis, to conduct a sensitivity analysis for RMI. All variables used in this study and those created were also used in the MI. Five datasets were created, and the number of iterations performed was also five. The predictive mean matching (PMM) algorithm was used as the imputation algorithm. The results were pooled using Rubin’s rules for proper uncertainty estimation.

Categorical variables are presented as frequencies and percentages. The continuous variables were mostly reported in terms of the mean, SD, min, max, and Confidence Interval (CI). Descriptive statistics, Chi-square test, Fisher’s exact test, independent samples Welch’s t-test, Mann-Whitney U test, one-way Welch’s Analysis of Variance (ANOVA), one-way Kruskal-Wallis test, and Spearman’s correlation were used as analysis methods in the univariate and bivariate analyses. In all the statistical analyses, a p-value < 0.05 was considered statistically significant.

### Ethical considerations

This study obtained ethical approval from the ethics committee at Åbo Akademi University (registration number: 36/2024) on November 1, 2024, and a research permit from the regional wellbeing services county in Finland (registration number: 6731/13.01/2024) was issued on December 5, 2024. This study was conducted by following the research guidelines published by the Finnish National Board of Research Integrity (TENK) [41], the General Data Protection Regulation [42], and the Declaration of Helsinki [43]. All participants gave written consent for participating in the study (which was confidential and voluntary) and the publication of the final results. An anonymised code was created for each pair of patient surveys and APN short questionnaires, which was only used for the data analysis. To ensure patient anonymity, the link cannot be traced to any individual participant. APNs were informed not to share their identification numbers with others to protect their anonymity. Only the first and fourth authors have access to the final APNs’ name list.

## Results

### Demographics of APN and patient participants

The demographic characteristics of the APN participants, as shown in Table 1, indicate that 25 APNs participated in this study. More than half of the APNs had Swedish as their native language.

**Table 1:** Characteristics of three APN groups (*n* = 25) working in primary care

Characteristics	Nurse prescriber (*n* = 13) *n* (%)	Nurse Practitioner (*n* = 5) *n* (%)	Nurse Practitioner with prescriptive authority (*n* = 7) *n* (%)	Total *n* (%)	*p*-value
**Gender** ^**1**^					
Woman	13 (100%)	5 (100%)	7 (100%)	25 (100%)	
**Age**					0.380^a^
31–40 years old	4 (30.8%)	1 (20.0%)	0 (0.0%)	5 (20.0%)	
41–50 years old	3 (23.1%)	3 (60.0%)	4 (57.1%)	10 (40.0%)	
51–60 years old	6 (46.2%)	1 (20.0%)	3 (42.9%)	10 (40.0%)	
Total n (%)	13 (100%)	5 (100%)	7 (100%)	25 (100%)	
**Native language**					0.002^b^
Finnish	9 (69.2%)	0 (0.0%)	0 (0.0%)	9 (36.0%)	
Swedish	4 (30.8%)	5 (100.0%)	7 (100.0%)	16 (64.0%)	
Total n (%)	13 (100%)	5 (100%)	7 (100%)	25 (100%)	

Note: 1 = All APNs were female. a = One-way Welch’s ANOVA. b=Independent samples Chi-square test of association

The characteristics of the patient participants are shown in Table 2; female patients were dominant. Half of the patients consulted APNs for acute health problems, while others visited APNs for long-term health problems or follow-up/ health check-ups. Nearly 60% of the patient participants had Swedish as their native language. However, no patients self-assessed their overall health as very poor, and nearly 75% considered their overall health as good or even better.

**Table 2:** Characteristics of patients (*n* = 425) consulting APNs and participating in this study

Characteristics	Acute health problem *n* (%)	Long-term health problem *n* (%)	Follow-up/health check-up *n* (%)	Total *n* (%)	*p*-value^a^
Gender (m* 10)					0.013
Man	62 (29.4%)	31 (38.3%)	59 (48.0%)	152 (36.6%)	
Woman	146 (69.2%)	50 (61.7%)	63 (51.2%)	259 (62.4%)	
Do not want to tell	3 (1.4%)	0 (0.0%)	1 (0.8%)	4 (1.0%)	
Total n (%)	211 (100%)	81 (100%)	123 (100%)	415 (100%)	
**Native languages**					0.006
Finnish	104 (47.9%)	28 (34.1%)	40 (31.7%)	172 (40.5%)	
Swedish	113 (52.1%)	54 (65.9%)	86 (68.3%)	253 (59.5%)	
Total n (%)	217 (100%)	82 (100%)	126 (100%)	425 (100%)	
**Patient language vs. APN language**					0.023
Same language	163 (75.1%)	69 (84.1%)	109 (86.5%)	341 (80.2%)	
Different languages	54 (24.9%)	13 (15.9%)	17 (13.5%)	84 (19.8%)	
Total n (%)	217 (100%)	82 (100%)	126 (100%)	425 (100%)	
Age groups (m*23)					< 0.001
0–17 years old	42 (20.9%)	7 (8.9%)	0 (0.0%)	49 (12.2%)	
18–65 years old	120 (59.7%)	29 (36.7%)	41 (33.6%)	190 (47.3%)	
> 65 years old	39 (19.4%)	43 (54.4%)	81 (66.4%)	163 (40.5%)	
Total n (%)	201 (100%)	79 (100%)	122 (100%)	402 (100%)	
Patient’s overall health status (m*13)					< 0.001
Excellent	36 (17.2%)	2 (2.5%)	8 (6.5%)	46 (11.2%)	
Very good	69 (33.0%)	18 (22.5%)	24 (19.5%)	111 (26.9%)	
Good	73 (34.9%)	28 (35.0%)	50 (40.7%)	151 (36.7%)	
Fair	28 (13.4%)	25 (31.3%)	39 (31.7%)	92 (22.3%)	
Poor	3 (1.4%)	7 (8.8%)	2 (1.6%)	12 (2.9%)	
Very poor	0 (0.0%)	0 (0.0%)	0 (0.0%)	0 (0.0%)	
Total n (%)	209 (100%)	80 (100%)	123 (100%)	412 (100%)	

Note: a= Independent samples Chi-square test of association. m*=missing

### Comparison of patient visits across APN groups

All APN groups cared for patients with both acute and long-term health problems; however, NPs with prescriptive authority and nurse prescribers had a lot more acute patients (Table 3). NPs reported a greater need to consult physicians for their patients than NPs with prescriptive authority and nurse prescribers. More than half of the patients went directly home after consulting APNs. Nurse prescribers and NPs with prescriptive authority had quite similar average visit times (about 30 min). NPs spent at least 10 more minutes on the average patient visit time than the other two groups and also had significantly more administrative time per patient.

**Table 3:** Overview of all patient visits (*n* = 483) to APN clinics in primary care

Characteristics	Nurse prescribers *n* (%)	Nurse Practitioners *n* (%)	Nurse Practitioners with prescriptive authority *n* (%)	Total *n* (%)	*p*-value
**Visit reasons**					0.021^a^
Acute health problem	137 (55.5%)	39 (40.6%)	71 (54.6%)	257 (53.2%)	
Long-term health problem	44 (17.8%)	14 (14.6%)	33 (25.4%)	91 (18.8%)	
Follow-up/ health check-up	66 (26.7%)	43 (44.8%)	26 (20.0%)	135 (28.0%)	
Total n (%)	247 (100%)	96 (100%)	130 (100%)	483 (100%)	
APNs’ need to consult physicians regarding patient visits (m*2)					< .001^a^
No	160 (65.0%)	38 (35.8%)	87 (67.4%)	285 (59.3%)	
Yes	86 (35.0%)	68 (64.2%)	42 (32.6%)	196 (40.7%)	
Total n (%)	246 (100%)	106 (100%)	129 (100%)	481 (100%)	
Post-consultation care pathways (m*1)					< .001^a^
Direct to the physician	34 (13.8%)	3 (2.9%)	8 (6.2%)	45 (9.3%)	
Direct home without a new appointment	146 (59.1%)	37 (35.2%)	64 (49.2%)	247 (51.2%)	
Direct home, new appointment with an APN	15 (6.1%)	18 (17.1%)	8 (6.2%)	41 (8.5%)	
Direct home, new appointment with the physician	24 (9.7%)	12 (11.4%)	33 (25.4%)	69 (14.3%)	
Follow-up with an APN through telephone or video conference	28 (11.3%)	35 (33.3%)	17 (13.1%)	80 (16.6%)	
Total n (%)	247 (100%)	105 (100%)	130 (100%)	482 (100%)	
**The length of consultation with APNs**	*n* = 247	*n* = 106	*n* = 130	*n* = 483	< .001^b^
Mean	29.4	44.7	32.4	33.6	
SD	14.1	16.2	16.1	16.2	
Min	6.0	10.0	7.0	6.0	
Max	70.0	90.0	90.0	90.0	
The length of administrative time (m*16)	*n* = 244	*n* = 105	*n* = 118	*n* = 467	< .001^b^
Mean	10.7	16.6	9.8	11.8	
SD	7.3	11.0	6.5	8.5	
Min	2.0	5.0	0.0	0.0	
Max	45.0	55.0	37.0	55.0	

Note: a= Independent samples Chi-square test of association. b = one-way Kruskal-Wallis’ test. m*= missing

### Comparison of parameters among the original dataset, RMI dataset, and MI dataset

A comparison of the parameters among the original dataset, RMI dataset, and MI dataset is shown in Table 4. The means and SDs of the variables were quite similar in all three groups. Although there was a slight change in the means and SDs with MI, these results were assessed as similar to RMI.

**Table 4:** Comparison of parameters among the original dataset, RMI dataset, and MI dataset (*n* = 425)

Variables	Parameter	Original	RMI	MI
Total score of PESS-ANP	Mean	81.4	81.4	80.9
	SD	7.4	7.4	7.6
Total score of patient satisfaction	Mean	70.4	70.4	70.3
	SD	6.3	6.3	6.2
Total score of patient enablement	Mean	10.8	10.8	10.6
	SD	2.8	2.8	2.9

Note: RMI = Row mean imputation. MI= multiple imputation

### Patient experiences of consulting APNs in primary care

An overview of patient experiences regarding their consultations with APNs is shown in Table 5. The results revealed that patients had high satisfaction and enablement after APN consultations. In comparison with the mean among items for patient satisfaction, items 1, 3, and 11–14 had the highest mean value (4.78–4.82). In the patient enablement items, the mean value of most items was close, except for the mean value of item 18, which was slightly lower than that of the other items.

**Table 5:** Overview of PESS-ANP results, including patient satisfaction and enablement after RMI (*n* = 425)

PESS-ANP items	Mean	Median	SD
**Patient satisfaction**			
1. The nurse was understanding of my personal health concerns.	4.78	5	0.46
2. The nurse gave me encouragement in regard to my health problem.	4.63	5	0.60
3. I felt comfortable to ask the nurse questions.	4.80	5	0.48
4. My questions were answered in an individual way.	4.71	5	0.55
5. I was included in decision-making.	4.45	5	0.70
6. I was included in the planning of my care.	4.48	5	0.68
7. The treatments I received were of a high quality.	4.66	5	0.57
8. Decisions regarding my health care were of high quality.	4.59	5	0.63
9. The nurse was available when I needed them.	4.63	5	0.63
10. The nurse appointment times were when I needed them.	4.64	5	0.65
11. The nurse spent enough time with me.	4.79	5	0.49
12. I was confident with the nurse’s skills.	4.82	5	0.46
13. The nurse was very professional.	4.80	5	0.47
14. Overall, I was satisfied with my health care.	4.78	5	0.47
15. The care I received from the nurse was of a high quality.	4.77	5	0.49
**Patient enablement**			
As a result of seeing the nurse, do you feel you are:			
16. able to understand your illness	2.17	2	0.63
17. able to cope with your illness	2.20	2	0.64
18. able to keep yourself healthy	2.06	2	0.68
19. confident about your health	2.18	2	0.68
20. able to help yourself	2.19	2	0.67

Note: Patient satisfaction items 1–15 have a scale of 1–5. Patient enablement items 16–20 have a scale of 1–3

The average total scores of the PESS-ANP, patient satisfaction, and patient enablement were similar across the three APN groups (Table 6). NPs with/without prescriptive authority were presented with slightly increased patient satisfaction compared to nurse prescribers, with about one more point added to their total scores.

**Table 6:** Comparison of patient experiences across APN groups after RMI (*n* = 425)

	Parameters	Nurse prescribers	Nurse practitioners	Nurse practitioners with prescriptive authority	Total	p-value^a^
Total score of PESS-ANP	Mean	81.1	81.5	82.0	81.44	0.561
	CI 95%	[80.1, 82.1]	[79.8,83.3]	[80.7, 83.3]	[80.7, 82.2]	
	SD	7.30	8.29	6.56	7.35	
Total score of patient satisfaction	Mean	69.9	71.7	70.9	70.4	0.272
	CI 95%	[69.0, 70.8]	[69.2, 72.2]	[70.0, 71.8]	[69.8, 70.9]	
	SD	6.38	7.29	4.93	6.25	
Total score of patient enablement	Mean	10.7	10.8	10.9	10.8	0.895
	CI 95%	[10.3, 11.1]	[10.3, 11.4]	[10.3, 11.4]	[10.5, 11.1]	
	SD	2.83	2.65	2.86	2.79	

Note: CI= Confidence interval. a = one-way Welch’s ANOVA

Additionally, the total PESS-ANP score was significantly higher in patients who spoke the same language as the APN (mean: 82.0, SD: 6.3) than in patients who spoke a different language (mean: 79.1, SD: 10.5; t (84.3) = -2.26, *p* = 0.026, independent samples Welch’s t test). Moreover, the patient’s overall health status was significantly correlated with the total scores of the PESS-ANP, with a coefficient of 0.164, a p-value of 0.001 (Spearman’s rho), and patient satisfaction and enablement, with a coefficient of 0.103 and p-value of 0.037, and a coefficient of 0.174 and p-value of < 0.001 (Spearman’s rho), respectively. However, other factors, such as patient gender, visit reasons, patient age, the length of consultation, and reasons for visit, had no significant bivariate association with patient experiences.

### Comparisons between the patient participation and non-participation groups

Of the 483 patients who participated in the study, only 425 (88.0%) completed the questionnaire. The following categories were overrepresented in the non-participation group: Finnish-speaking APNs, nurse prescribers, acute reasons for visits, and visiting a doctor immediately after the APN. In addition, the average visit times were also slightly shorter in the non-patient participation group than in the patient participation group (Table 7). Specifically, of the Finnish-speaking APNs (vs. Swedish-speaking), 91.4% were nurse prescribers (vs. 25.7% of Swedish-speaking APNs, *p* < 0.001, Fisher’s test), and 66.3% had patients with acute reasons for their visit (vs. 44.9%, *p* < 0.001, Fisher’s test). The average visit times were also slightly shorter at 27.3 min (vs. 37.5 min, *p* < 0.001, Welch’s test). However, all these categorical variables of nurses’ responses showed only incremental differences for total PESS scores, with nonsignificant p-values.

**Table 7:** Comparison of APN responses between the patient participation and non-participation groups

Variables	Patient groups		*p*-value
	Non-participation	Particiation	
Nurse group			0.356^a^
Nurse prescribers	35 (60.3%)	212 (49.9%)	
Nurse Practitioners	10 (17.2%)	96 (22.6%)	
Nurse Practitioners with prescriptive authority	13 (22.4%)	117 (27.5%)	
Total	58 (100%)	425 (100%)	
Nurse language			0.004^a^
Finnish	33 (56.9%)	154 (36.2%)	
Swedish	25 (43.1%)	271 (63.8%)	
Total	58 (100%)	425 (100%)	
Reason for visit			0.030^a^
Acute health problem	40 (69.0%)	217 (51.1%)	
Long-term health problem	9 (15.5%)	82 (19.3%)	
Follow-up/health check-up	9 (15.5%)	126 (29.6%)	
Total	58 (100%)	425 (100%)	
After visit			0.036^a^
Direct to the physician	11 (19.3%)	34 (8.0%)	
Direct home without a new appointment	29 (50.9%)	218 (51.3%)	
Direct home, new appointment with an APN	4 (7.0%)	37 (8.7%)	
Direct home, new appointment with the physician	9 (15.8%)	60 (14.1%)	
Follow-up with an APN through telephone or video	4 (7.0%)	76 (17.9%)	
Total	57 (100%)	425 (100%)	
Doctor consultation			0.776^a^
Yes	25 (43.1%)	171 (40.4%)	
No	33 (56.9%)	252 (59.6%)	
Total	58 (100%)	423 (100%)	
Visit time in minutes (mean)	27.6	34.4	< 0.001^b^
			0.001^c^
Administrative time in minutes (mean)	8.8	12.2	< 0.001^b^
			< 0.001^c^

Note: a= Fisher’s exact test; b= Welch’s t-test; c= Mann-Whitney U test

## Discussion

The findings indicated that APNs provided care to patients of all ages, addressing both acute and long-term condition needs. Patient participants highly rated their experience regarding APN consultations in primary care, showing high levels of satisfaction and enablement. The slight observed differences among total scores of the PESS-ANP, patient satisfaction, and enablement across the three APN groups (i.e. nurse prescribers, NPs, and NPs with prescriptive authority) were statistically nonsignificant. However, two potential factors that were found to be associated with patient experience were the patient’s overall health status and whether or not they had the same language as APNs.

In this study, the results showed that patients were enabled and highly satisfied with APN consultations. The surveys were distributed to patients who completed it immediately after consulting the APNs, which enabled them to accurately report their recent experiences. The findings regarding patient enablement are consistent with other studies utilising the PESS-ANP [21, 30]. In this vein, another study revealed that more than 80% (*n* = 65) of patients reported better or much better enablement after consulting the diabetes CNS virtually [44]. Furthermore, the fact that the patients expressed high satisfaction with APN consultations in this study is consistent with international studies showing that advanced practice nursing positively impacts patient satisfaction [5, 45–47]. In the Nordic region, these results were aligned with a Swedish primary care study in which patients were overall highly satisfied with APN-led care in locations where advanced practice nursing was implemented. In addition, patients were more satisfied when they were awared of APN roles and referred to APNs prior to their visits to healthcare centres [48].

According to the results, patient age and gender had no association with the PESS-ANP results. However, patients’ overall health status was positively correlated with the total score of patient experiences (i.e. patient satisfaction and patient enablement). Approximately three-quarters of the patients rated their overall health status at least at “good”. In contrast, one-quarter of the patients rated their overall health status as “fair” or “bad”, and no patient participant evaluated their overall health status as “very bad”. Patients with better overall health status scored higher on the PESS-ANP items. One study in Canada showed that a patient’s age, gender, education, and health status had no association with patient satisfaction [49]. Remarkably, the sample size was significantly smaller (*n* = 113), and the patient visit type was only acute care patients. In contrast, younger patients reported slightly higher patient satisfaction than older patients in a study from New Zealand, but gender did not have an influence here [50]. In summary, the findings from these studies differ from those of the present research regarding the association between patient experiences, overall health status, and age.

Language differences between patients and APNs significantly impact patient experience, suggesting that patients prefer consulting APNs who speak their native language. Communication has been considered a relevant healthcare-provider determinant in relation to patient satisfaction [51]. In fact, health services and care provided in the same language between the care provider and patients have been proven to increase patient satisfaction [52]. Improving access to language-appropriate health care services is essential for patients who speak different languages, especially in countries like the United States, which has a diverse and growing multilingual population [53]. Overall, communication is key to higher satisfaction levels in primary care, with studies reporting that the states of a patient’s health and patient ethnicity/language skills are significant in determining patient enablement [20].

The findings revealed that the patients presented with various health care problems to the APNs. Some APN participants had a more focused role, while 15 APNs consulted any health problems and any age group. One previous study [3] found similar results. In terms of APNs’ practice patterns, six APNs cared for patients with long-term conditions and also offered these patients follow-up and health assessments, while four APNs were mainly responsible for patients with acute health problems. The implementation of APN roles in Europe varies in different countries, and the size of the APN workforce is generally contingent upon patient needs and care delivery models [54]. In France, for instance, APNs usually take care of patients with long-term conditions or specialist care needs [55]. In contrast, APNs work in all care settings and serve a wider range of patient groups in the study region.Overall, the results of this study provide evidence of the implementation of advanced practice nursing from the patient’s perspective. Generally, the patients were highly satisfied and felt enabled with the care they received from the APNs. Language and the patient’s overall health status were identified as two relevant factors associated with patient experience including patient satisfaction and enablement in primary care.

### Limitations

First, as a cross-sectional study, only descriptive and correlated results of patients’ experiences about APN consultations have been presented. It is thus not possible to make any causal inferences about these relationships. Second, although the number of patient participants was relatively high, patients who were seen virtually by APNs were excluded from this study. Additionally, the self-reported data may include memory errors, as some patients took the surveys home and returned them later. Finally, the study covers only one wellbeing services county in Finland, which cannot represent the results of advanced practice nursing at a national level. However, the sample size was considered adequate for this analysis. Finally, this study is only the first patient of its kind about APN clinics in Finland; however, it can still provide evidence for the Nordic context or somewhere else where APN role development is still in its infancy stage.

Although there are several limitations, this study has addressed the bias of non-participation and missing data. RMI was chosen to deal with the missing data. Imputation has been regarded as a preferred method when handling missing data [56]. Mean imputation is a traditional approach for imputation and should be used to improve the data analysis [57]. Variation within items of the PESS-ANP in both sections in the same row was exceptionally low, which supported the selection of this method. According to Table 4, RMI was the more appropriate choice when compared with MI. While this study had a non-participation rate of just over 10%, the study design with two separate participant groups enabled a more detailed analysis of patient non-participation and the potential bias it may cause. Although there were some differences in the nurses’ responses between non-participating and participating patients, the total PESS-ANP scores, however, did not differ significantly across the various background variable groups. Therefore, this difference did not cause significant bias.

## Conclusion

This study provides valuable evidence about the patient experience of utilising care and services during APN consultations in Finland. On the one hand, it provides more information for policy makers and nurse leaders about patients’ perspectives regarding the experiences of advanced practice nursing in primary care. On the other hand, it may contribute to the legislative process of ensuring title protection and regulation for APN roles at a national level. More data on patient experiences and outcome studies of APN consultations are needed to promote APN roles in countries where these roles are still under development.

## Supplementary Information

Below is the link to the electronic supplementary material.

Supplementary Material 1

## Data Availability

The datasets analysed during the current study are not publicly available due to ethical and legal considerations regarding participants’ confidentiality and anonymity. However, the confidential data is available from the corresponding author on reasonable request.

## Declarations

### Authors’ information

Both LF and MR have significant research experience in advanced practice nursing. MR has extensive clinical experience as an NP in the UK. AR has a strong background in statistical analysis and quantitative research in the healthcare context. RH is a registered nurse in Finland and has three years of research experience in advanced practice nursing.

## Abbreviations

ANOVA: Analysis of Variance
APN: Advanced Practice Nurse
CI: Confidence interval
CNS: Clinical Nurse Specialist
CST: Client Satisfaction Tool
EQF: European Qualification Framework
MCAR: Missing completely at random
MI: Multiple imputation
mice: Multiple imputation by chained equation
NP: Nurse Practitioners
OECD: Organisation for Economic Co-operation and Development
PEI: Patient Enablement Instrument
PESS-ANP: Patient Enablement and Satisfaction Survey- Advanced Nurse Practitioners
PMM: predictive mean matching
RN: registered nurse
RMI: Row mean imputation
SD: Standard deviation
STROBE: STrenthing the Reporting of Observational studies in Epidemiology
TENK: Finnish National Board of Research Integrity

## References

[1] Fagerström LM. A caring advanced practice nursing model: theoretical perspectives and competency domains. Cham: Springer International Publishing; 2021.

[2] Mackavey C, Henderson C, Morris G. Empowering advanced practice nurses: a review of addressing global health needs. Ann Glob Health. 2025. 10.5334/aogh.4723. 40821623 10.5334/aogh.4723 PMC12352385

[3] Han R, Rogers M, Asplund S, Fagerström L. Nurse practitioners’ practice patterns in primary care in Finland: a descriptive qualitative study. Glob Qual Nurs Res. 2025. 10.1177/23333936251365612. 40842567 10.1177/23333936251365612 PMC12365369

[4] Htay M, Whitehead D. The effectiveness of the role of advanced nurse practitioners compared to physician-led or usual care: a systematic review. Int J Nurs Stud Adv. 2021. 10.1016/j.ijnsa.2021.100034. 38746729 10.1016/j.ijnsa.2021.100034 PMC11080477

[5] Woo BFY, Lee JXY, Tam WWS. The impact of the advanced practice nursing role on quality of care, clinical outcomes, patient satisfaction, and cost in the emergency and critical care settings: a systematic review. Hum Resour Health. 2017;15(1):63. 28893270 10.1186/s12960-017-0237-9 PMC5594520

[6] McMenamin A, Turi E, Schlak A, Poghosyan L. A systematic review of outcomes related to nurse practitioner-delivered primary care for multiple chronic conditions. Med Care Res Rev. 2023. 10.1177/10775587231186720. 37438917 10.1177/10775587231186720 PMC10784406

[7] Pellet J, Levasseur I, Mabire C. Les infirmiers-ères de pratique spécialisée pour renforcer les soins de première ligne. Rev Med Suisse. 2024. 10.53738/REVMED.2024.20.898-2.2335. 39663968 10.53738/REVMED.2024.20.898-2.2335

[8] International Council of Nurses. Guidelines on advanced practice nursing 2020. 2020. https://www.icn.ch/resources/publications-and-reports/guidelines-advanced-practice-nursing-2020. Accessed 6 Jan 2026.

[9] Advanced practice nursing in primary care in OECD countries: recent developments and persisting implementation challenges. OECD Health Working Papers; vol. 165. Report No: 165. 2024. https://www.oecd.org/en/publications/advanced-practice-nursing-in-primary-care-in-oecd-countries_8e10af16-en.html. Accessed 30 Dec 2025.

[10] Kleinpell R, Kapu AN, Woo B, Wentao Z. Nurse practitioner outcomes evaluation. In: Thomas SL, Rowles JS, editors. Nurse practitioners and nurse anesthetists: the evolution of the global roles. Cham: Springer International Publishing; 2023. pp. 119–27.

[11] Kleinpell R. Artificial intelligence knows the value of nurse practitioners–why can’t other humans? J Am Assoc Nurse Pract. 2023;35(10):599. 37751534 10.1097/JXX.0000000000000898

[12] Kleinpell RM, editor. Outcome assessment in advanced practice nursing. 5th ed. New York: Springer Publishing Company; 2021.

[13] van Erp RMA, van Doorn AL, van den Brink GT, Peters JWB, Laurant MGH, van Vught AJ. Physician assistants and nurse practitioners in primary care plus: a systematic review. Int J Integr Care. 2021;21(1):6. 33613138 10.5334/ijic.5485 PMC7879997

[14] Nahari A, Moafa H, Almotairy MM, Hakamy E, Alhamed A, Alkhaifi S, et al. Lived experiences of nurse practitioners in Saudi Arabia: a phenomenological study. BMC Nurs. 2025;24(1):510. 40355850 10.1186/s12912-025-03137-2 PMC12067684

[15] Weinman J. Doctor–patient interaction: psychosocial aspects. In: Smelser NJ, Baltes PB, editors. International encyclopedia of the social & behavioral sciences. Oxford: Elsevier; 2001. pp. 3816–21.

[16] Paddison CAM, Abel GA, Roland MO, Elliott MN, Lyratzopoulos G, Campbell JL. Drivers of overall satisfaction with primary care: evidence from the English General Practice Patient Survey. Health Expect. 2015;18(5):1081–92. 23721257 10.1111/hex.12081 PMC5060812

[17] Dilworth S, Ball J, Giles M, Lackay L, Dahlstrom C, Fahy M, et al. Patient acceptability and satisfaction with the rural emergency department nurse practitioner model of care (RED-NP MoC). Australas Emerg Care. 2025;28(4):268–73. 40425370 10.1016/j.auec.2025.05.002

[18] Deay D, O’Keefe O, Byrne M, McBrien B, McCabe A. Evaluation of an advanced nurse practitioner led emergency rapid assessment and treatment service. Int Emerg Nurs. 2025. 10.1016/j.ienj.2025.101684. 41014775 10.1016/j.ienj.2025.101684

[19] Baumgartner E, Elicin O, Mueller SA, Giger R, Spichiger E. Patient experiences before and after the implementation of advanced nursing practice for head and neck cancer. Clin J Oncol Nurs. 2025. 10.1188/25.CJON.E146-E157. 40986766 10.1188/25.CJON.E146-E157 PMC12463158

[20] Tolvanen E, Koskela TH, Helminen M, Kosunen E. Patient Enablement After a Single Appointment With a GP: Analysis of Finnish QUALICOPC Data. J Prim Care Community Health. 2017;8(4):213–20. 28911251 10.1177/2150131917730211 PMC5932738

[21] Frost J, Currie MJ, Cruickshank M, Northam H. Using the lens of enablement to explore patients’ experiences of Nurse Practitioner care in the Primary Health Care setting. Collegian. 2018;25(2):193–9.

[22] McConkey R, Murphy L, Kelly T, Dalton R, Rooney G, Coy D, et al. Patient-Reported Enablement After Consultation With Advanced Nurse Practitioners: A Cross-Sectional Study. J Nurse Pract. 2023;19(9):104764.

[23] Barratt J, Thomas N. Nurse practitioner consultations in primary health care: a case study-based survey of patients’ pre-consultation expectations, and post-consultation satisfaction and enablement. Prim Health Care Res Dev. 2018;20:e36. 30012232 10.1017/S1463423618000415 PMC6536762

[24] Frost JS, Currie MJ, Cruickshank M, Northam HL. Viewing Nurse Practitioners’ Perceptions of Patient Care Through the Lens of Enablement. J Nurse Pract. 2017;13(8):570–6.

[25] The Finnish Nurses Association Expert Working Group on APN. Advanced practice nursing in Finland – a clinical nursing career model. 2023. https://sairaanhoitajat.fi/wp-content/uploads/2024/09/APN-report-2023-English.pdf. Accessed 10 Jan 2026.

[26] Tuomikoski AM, Flinkman M, Sulosaari V, Suutarla A, Jokiniemi K. Laajavastuisen hoitotyön asiantuntijuus ja sen tutkimus Suomessa: kartoittava katsaus: APN-kartoittava katsaus. Hoitotiede. 2024;36(1):82–101.

[27] von Elm E, Altman DG, Egger M, Pocock SJ, Gøtzsche PC, Vandenbroucke JP. The strengthening the reporting of observational studies in epidemiology (STROBE) statement: guidelines for reporting observational studies. Lancet. 2007;370(9596):1453–7. 18064739 10.1016/S0140-6736(07)61602-X

[28] O’Reilly D, Brady AM, Bryant-Lukosius D, Varley J, Daly L, Cotter P, et al. Patient-reported experiences of consultation with an advanced nurse practitioner: factor structure and reliability analysis of the patient enablement and satisfaction survey. J Adv Nurs. 2021;77(10):4279–89. 34449917 10.1111/jan.15026

[29] Desborough J, Banfield M, Parker R. A tool to evaluate patients’ experiences of nursing care in Australian general practice: development of the Patient Enablement and Satisfaction Survey. Aust J Prim Health. 2014;20(2):209–15. 23469805 10.1071/PY12121

[30] Bear M, Bowers C. Using a nursing framework to measure client satisfaction at a nurse-managed clinic. Public Health Nurs Boston Mass. 1998;15(1):50–9. 10.1111/j.1525-1446.1998.tb00321.x 9503954

[31] Howie JG, Heaney DJ, Maxwell M, Walker JJ. A comparison of a Patient Enablement Instrument (PEI) against two established satisfaction scales as an outcome measure of primary care consultations. Fam Pract. 1998;15(2):165–71. 9613486 10.1093/fampra/15.2.165

[32] Brislin RW. Back-Translation for Cross-Cultural Research. J Cross-Cult Psychol. 1970;1(3):185–216.

[33] Maneesriwongul W, Dixon JK. Instrument translation process: a methods review. J Adv Nurs. 2004;48(2):175–86. 15369498 10.1111/j.1365-2648.2004.03185.x

[34] Jamovi. jamovi – open statistical software for the desktop and cloud. 2025. https://www.jamovi.org/. Accessed 3 Nov 2025.

[35] R Core Team. R: the R Project for Statistical Computing. 2025. https://www.r-project.org/. Accessed 28 Nov 2025.

[36] Little RJA. A Test of Missing Completely at Random for Multivariate Data with Missing Values. J Am Stat Assoc. 1988;83(404):1198–202.

[37] Tierney N, Cook D. Expanding tidy data principles to facilitate missing data exploration, visualization and assessment of imputations. J Stat Softw. 2023;105:1–31.

[38] van Buuren S, Groothuis-Oudshoorn K, Vink G, Schouten R, Robitzsch A, Rockenschaub P, et al. Mice: multivariate imputation by chained equations. 2025. https://cran.r-project.org/web/packages/mice/index.html. Accessed 3 Nov 2025.

[39] Children’s and youths’ rights and obligations. 2026. https://www.infofinland.fi/en/family/children/childrens-and-youths-rights-and-obligations. Accessed 1 Jan 2026.

[40] R: mean imputation. 2025. https://search.r-project.org/CRAN/refmans/missMethods/html/impute_mean.html. Accessed 3 Nov 2025.

[41] Finnish National Board on Research Integrity TENK. The Finnish code of conduct for research integrity and procedures for handling alleged violations of research integrity in Finland 2023. 2023. https://tenk.fi/sites/default/files/2023-05/RI_Guidelines_2023.pdf. Accessed 1 Jan 2026.

[42] General Data Protection Regulation (GDPR) – official legal text. 2024. https://gdpr-info.eu/. Accessed 27 Dec 2025.

[43] World Medical Association. WMA Declaration of Helsinki – ethical principles for medical research involving human participants. 2025. https://www.wma.net/policies-post/wma-declaration-of-helsinki/. Accessed 9 Dec 2025. 10.1001/jama.2024.21972 39425955

[44] McCabe C, McCann M, Connolly D, McGrath J, Begley J, Ball JC, et al. Impact of unscheduled nurse-led virtual care for people with diabetes on nursing practices and patient satisfaction. Br J Nurs. 2024;33(5):236–41. 38446518 10.12968/bjon.2024.33.5.236

[45] Roche TE, Gardner G, Jack L. The effectiveness of emergency nurse practitioner service in the management of patients presenting to rural hospitals with chest pain: a multisite prospective longitudinal nested cohort study. BMC Health Serv Res. 2017;17(1):445. 28655309 10.1186/s12913-017-2395-9 PMC5488347

[46] Dinh M, Walker A, Parameswaran A, Enright N. Evaluating the quality of care delivered by an emergency department fast track unit with both nurse practitioners and doctors. Australas Emerg Nurs J. 2012;15(4):188–94. 23217651 10.1016/j.aenj.2012.09.001

[47] Greenbank K, Hemingway S, Thiyagesh S, Stephenson J. Service user and carer experiences of the advanced nurse practitioner role in a memory assessment team. Br J Nurs. 2020;29(16):960–7. 32901537 10.12968/bjon.2020.29.16.960

[48] Bergman K, Perhed U, Eriksson I, Lindblad U, Fagerström L. Patients’ satisfaction with the care offered by advanced practice nurses: A new role in Swedish primary care. Int J Nurs Pract. 2013;19(3):326–33. 23730865 10.1111/ijn.12072

[49] Thrasher C, Purc-Stephenson R. Patient satisfaction with nurse practitioner care in emergency departments in Canada. J Am Acad Nurse Pract. 2008;20(5):231–7. 18460162 10.1111/j.1745-7599.2008.00312.x

[50] Jo Gagan M, Maybee P. Patient satisfaction with Nurse Practitioner care in primary care settings. Aust J Adv Nurs. 2011;28(4):12–9.

[51] Akthar N, Nayak S, Pai PY. Determinants of patient satisfaction in Asia: Evidence from systematic review of literature. Clin Epidemiol Glob Health. 2023;23:101393.

[52] Diamond L, Izquierdo K, Canfield D, Matsoukas K, Gany F. A systematic review of the impact of patient–physician non-English language concordance on quality of care and outcomes. J Gen Intern Med. 2019;34(8):1591–606. 31147980 10.1007/s11606-019-04847-5 PMC6667611

[53] Ortega P, Shin TM, Martínez GA. Rethinking the term limited English proficiency to improve language-appropriate healthcare for all. J Immigr Minor Health. 2022;24(3):799–805. 34328602 10.1007/s10903-021-01257-w PMC8323079

[54] Unsworth J, Greene K, Ali P, Lillebø G, Mazilu DC. Advanced practice nurse roles in Europe: implementation challenges, progress and lessons learnt. Int Nurs Rev. 2024. 10.1111/inr.12800. 36094718 10.1111/inr.12800

[55] Colson S, Schwingrouber J, Evans C, Roman C, Bourriquen M, Lucas G, et al. The creation and implementation of advanced practice nursing in France: Experiences from the field. Int Nurs Rev. 2021;68(3):412–9. 34152009 10.1111/inr.12684

[56] Alam S, Ayub MS, Arora S, Khan MA. An investigation of the imputation techniques for missing values in ordinal data enhancing clustering and classification analysis validity. Decis Anal J. 2023. 10.1016/j.dajour.2023.100341.

[57] Huisman M. Imputation of missing network data: some simple procedures. In: Alhajj R, Rokne J, editors. Encyclopedia of social network analysis and mining. New York, NY: Springer; 2014. pp. 707–15.

