# Supplementary material for: Patient experiences of consulting advanced practice nurses in primary care: a cross-sectional study
Source: BMC Health Serv Res. 2026 Apr 11;26:727. doi: 10.1186/s12913-026-14511-4 (PMC13196175; doi:10.1186/s12913-026-14511-4)
Supplement: Supplementary file 1 — Supplementary Material 1 [file 12913_2026_14511_MOESM1_ESM.pdf]

A short questionnaire regarding patient visits- for Advanced Practice Nurses working in primary care

*****Please complete this section only once at the beginning of the survey.**

Part 1 Background information

1.Age____

2.Sex:

- ☐ Male
- ☐ Female
- ☐ Do not want to tell

3.Education ___________________________

*****Please complete this section after each patient visit.**

Part 2 The following questions regarding patient visits:

1.The length of the consultation time (in minutes): ________

The administrative time after the consultation (in minutes): ________

2. Select the reason for patient consultation:

- ☐ Acute health problems
- ☐ Long-term health problems
- ☐ Follow-up/ health check-up

Please describe these problems with a few words:

____

3. Did you need to consult a physician during or immediately after the patient visit?

- ☐ Yes
- ☐ No

4. Where was the patient referred after the visit?

- ☐ Directly to the physician
- ☐ Directly home without a new appointment
- ☐ Directly home with a new appointment with an APN (follow-up)
- ☐ Directly home with a new appointment with the physician
- ☐ A follow-up with the APN via telephone or videoconference
